# Heat-Insulated Regenerated Fibers with UV Resistance: Silk Fibroin/Al_2_O_3_ Nanoparticles

**DOI:** 10.3390/molecules29092023

**Published:** 2024-04-27

**Authors:** Jianjun Guo, Song Lu, Yi Zhou, Yuanyuan Yang, Xiaoxian Yao, Guohua Wu

**Affiliations:** 1 College of Agriculture, Anshun University, Anshun 561000, China; jianjunguo868@163.com (J.G.); zhouyi199103@163.com (Y.Z.); yaoxiaoxian2002@163.com (X.Y.); 2 College of Biotechnology, Jiangsu University of Science and Technology, Zhenjiang 212100, China; songlu0414@foxmail.com (S.L.); yangyuanyuansmooth@foxmail.com (Y.Y.)

**Keywords:** smart textile, Al_2_O_3_ nanoparticles, regenerated silk fibroin, heat insulation, UV resistance

## Abstract

The various wastes generated by silkworm silk textiles that are no longer in use are increasing, which is causing considerable waste and contamination. This issue has attracted widespread attention in countries that use a lot of silk. Therefore, enhancing the mechanical properties of regenerated silk fibroin (RSF) and enriching the function of silk are important directions to expand the comprehensive utilization of silk products. In this paper, the preparation of RSF/Al_2_O_3_ nanoparticles (NPs) hybrid fiber with different Al_2_O_3_ NPs contents by wet spinning and its novel performance are reported. It was found that the RSF/Al_2_O_3_ NPs hybrid fiber was a multifunctional fiber material with thermal insulation and UV resistance. Natural light tests showed that the temperature rise rate of RSF/Al_2_O_3_ NPs hybrid fibers was slower than that of RSF fibers, and the average temperature rose from 29.1 °C to about 35.4 °C in 15 min, while RSF fibers could rise to about 40.1 °C. UV absorption tests showed that the hybrid fiber was resistant to UV radiation. Furthermore, the addition of Al_2_O_3_ NPs may improve the mechanical properties of the hybrid fibers. This was because the blending of Al_2_O_3_ NPs promoted the self-assembly of β-sheets in the RSF reaction mixture in a dose-dependent manner, which was manifested as the RSF/Al_2_O_3_ NPs hybrid fibers had more β-sheets, crystallinity, and a smaller crystal size. In addition, RSF/Al_2_O_3_ NPs hybrid fibers had good biocompatibility and durability in micro-alkaline sweat environments. The above performance makes the RSF/Al_2_O_3_ NPs hybrid fibers promising candidates for application in heat-insulating and UV-resistant fabrics as well as military clothing.

## 1. Introduction

Natural protein fiber has a wide range of applications in many aspects [1]. Silk fibroin is a natural protein secreted by insects, such as silk. It has excellent performance characteristics, such as lightness, softness, durability, and high tensile strength. These characteristics make silk fibroin widely used in the field of textiles [2,3]. However, the poor thermal insulation and UV resistance of silk fibroin limit its application in some special application scenarios [4,5]. Therefore, researchers have begun to focus on how to improve the thermal insulation and UV resistance of silk fibroin [6,7,8,9].

Through an in-depth study of the structure and characteristics of silk fibroin, researchers have found that the heat insulation and UV resistance of silk fibroin can be enhanced by changing the composition, structure, and treatment of silk fibroin. For example, researchers altered the composition and proportion of silk fibroin by changing the feeding methods, regulating the crystal structure of silk fibroin fiber, and improving its UV resistance [10].

In addition, the researchers also used chemical modification and functional addition methods to introduce materials with thermal insulation properties into silk fibroin to enhance its thermal insulation performance [11]. Therefore, the research background of silk fibroin in heat insulation and ultraviolet resistance is mainly to improve and enhance its performance to meet the needs of material properties in different application fields. These studies help to develop more functional and diverse silk fibroin textiles and expand their application.

**Figure 1:**
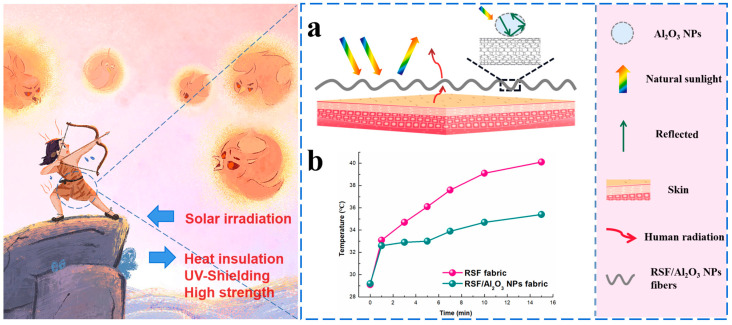
Schematic illustration of RSF/Al_2_O_3_ NPs hybrid fibers with heat insulation and UV resistance. (**a**) Diagram of the photothermal conversion process with RSF/Al_2_O_3_ NPs fabric. (**b**) Temperature change of RSF fabric and RSF/Al_2_O_3_ NPs fabric under natural sunlight for 15 min.

α-phase nano-alumina (α-Al_2_O_3_ NPs) is a kind of high-temperature stable nanoparticle [12], with excellent thermal conductivity [13,14] and a high specific surface area [15,16], which makes it have potential in the fields of thermal insulation and UV resistance. The research background of α-Al_2_O_3_ NPs in thermal insulation and UV resistance mainly involves the following aspects: It has good stability at high temperatures and can maintain its thermal insulation performance in high temperature environments [17]. Ji et al. successfully prepared alumina aerogel nanosheets with silica sol as a high-temperature binder with excellent ultra-high temperature and thermal insulation. Even after calcination at up to 1600 °C, it exhibits excellent thermal and chemical stability [18]. Al_2_O_3_ NPs co-catalyst could act as a multifunctional finishing material. The flame-retardant property of the fabric, besides wrinkle resistance, was improved [19]. It was found that an approximately 2-nm-thick Al_2_O_3_ insulating layer was introduced between ZnO NWs and Au NPs, causing the quenching phenomenon to disappear [20]. As a material with excellent thermal insulation and ultraviolet resistance, α-Al_2_O_3_ NPs had broad application prospects in the fields of textiles, construction, and so on. In this study, α-Al_2_O_3_ NPs were used to modify RSF, and RSF/Al_2_O_3_ NPs hybrid fibers were prepared by wet spinning. The structure and properties of RSF were modified by Al_2_O_3_ NPs in order to prepare a high-toughness RSF/Al_2_O_3_ NPs hybrid fiber with heat insulation and ultraviolet resistance under sunlight (Figure 1).

## 2. Results and Discussion

### 2.1. Morphology of RSF/Al_2_O_3_ NPs Hybrid Fibers

**Figure 2:**
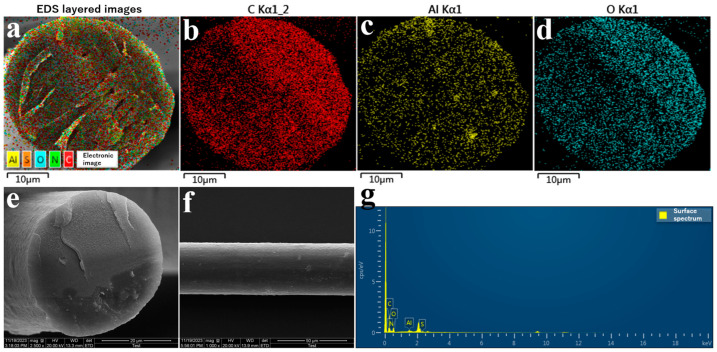
EDS images of 0.8 wt% RSF/Al_2_O_3_ NPs hybrid fibers (**a**–**d**,**g**); SEM images of 0.8 wt% RSF/Al_2_O_3_ NPs hybrid fibers (**e**) Internal structure; (**f**) Surface structure.

Scanning electron microscopy with energy dispersive X-ray spectroscopy (SEM-EDS) component spectrum of the 0.8 wt% RSF/Al_2_O_3_ NPs hybrid fibers is shown in Figure 2a–d. It illustrates the distribution of Al element in the RSF/Al_2_O_3_ NPs hybrid fibers, with the Al evenly distributed throughout, indicating an effective and uniform distribution of the Al_2_O_3_ NPs in the RSF/Al_2_O_3_ NPs hybrid fibers. As illustrated in Figure 2e,f, the surfaces of 0.8 wt% RSF/Al_2_O_3_ NPs hybrid fibers were shown to be smooth and homogeneous, free of any cavities or fractures in the interior structure. Therefore, adding Al_2_O_3_ NPs to RSF fibers did not affect their initial structure or qualities, and it was these traits that contributed to the excellent mechanical performance of the RSF/Al_2_O_3_ NPs hybrid. We discovered that the fibers had an average diameter of 37.55 ± 0.62 μm. The total spectrum of elements in the 0.8 wt% RSF/Al_2_O_3_ NPs hybrid fibers is shown in Figure 2g. It can be observed that all elements were detected in the hybrid fibers, confirming the successful distribution of Al_2_O_3_ NPs. Among them, in the 0.8 wt% RSF/Al_2_O_3_ NPs hybrid fibers, the elements C, N, O, S, and Al made up 42.14%, 20.70%, 36.23%, 0.26%, and 0.67% of the total, respectively (Figure 2g).

### 2.2. Mechanical Properties of RSF/Al_2_O_3_ NPs Hybrid Fibers

**Figure 3:**
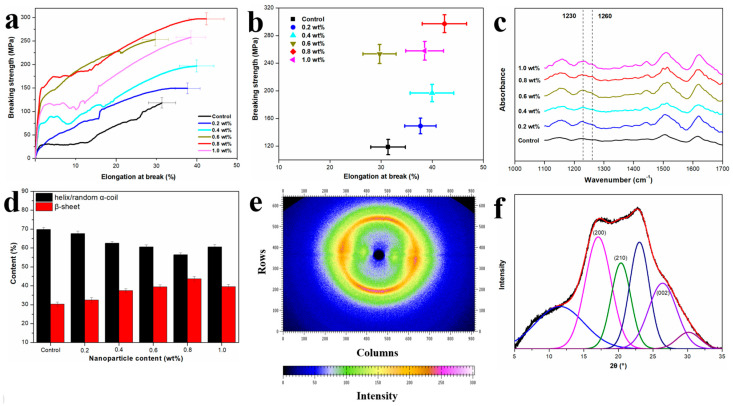
(**a**,**b**) Stress-strain curves of RSF and RSF/Al_2_O_3_ NPs hybrid fibers with different concentrations. (**c**) Infrared spectra of RSF and RSF/Al_2_O_3_ NPs hybrid fibers with different concentrations. (**d**) Secondary structure content of RSF and RSF/Al_2_O_3_ NPs hybrid fibers with different concentrations. (**e**) 2D-WAXD pattern of 0.8 wt% RSF/Al_2_O_3_ NPs hybrid fibers. (**f**) WAXD pattern deconvolution of 0.8 wt% RSF/Al_2_O_3_ NPs hybrid fibers.

Figure 3a shows the stress-strain curves of RSF fibers and RSF/Al_2_O_3_ NPs hybrid fibers. The 0.8 wt% RSF/Al_2_O_3_ NPs hybrid fibers exhibited the best mechanical properties when compared with RSF fibers. Its breaking strength (300.04 ± 17.25 MPa) and elongation at break (40.88 ± 12.45%) are higher than those of the blank group (Figure 3a), respectively, indicating that Al_2_O_3_ NPs could enhance the mechanical properties of the hybrid fibers to a certain extent. This phenomenon suggested that Al_2_O_3_ NPs had a significant toughening effect on the RSF fibers.

Further analysis of the effect of Al_2_O_3_ NPs on the mechanical properties of RSF/Al_2_O_3_ NPs hybrid fibers was conducted by plotting the breaking strength and elongation at the break curve of RSF/Al_2_O_3_ NPs hybrid fibers with varying Al_2_O_3_ NPs concentrations (Figure 3b). Results indicated that with increasing Al_2_O_3_ NPs content, both the breaking strength and elongation at break initially increased, reaching a maximum at 0.8 wt% Al_2_O_3_ NPs, before decreasing. Notably, the increase in breaking strength was more pronounced compared with elongation at break. In summary, the excellent mechanical properties make RSF/Al_2_O_3_ NPs hybrid fibers potential fibers for heat-insulated fabrics.

### 2.3. Structure Analysis of RSF/Al_2_O_3_ NPs Hybrid Fibers

The secondary structures of silk were strongly associated with their mechanical qualities, such as tensile modulus, elongation at break, and breaking strength [21]. To elucidate the mechanism underlying the enhanced mechanical properties, the secondary structure and crystal structure of RSF fibers and RSF/Al_2_O_3_ NPs hybrid fibers were investigated by fourier transform infrared spectroscopy (FTIR) and wide-angle X-ray diffraction (WAXD) [22]. As illustrated in Figure 3c, both RSF fibers and RSF/Al_2_O_3_ NPs hybrid fibers show peak locations almost identical to their corresponding FTIR spectra. It suggests that Al_2_O_3_ NPs have no impact on the secondary structural components of RSF fibers.

We de-convoluted the amide III spectral area of both the RSF fibers and RSF/Al_2_O_3_ NPs hybrid fibers for probing into what was in the secondary structural components of both materials [23]. The relative content of α-helix/random coil (1230 cm^−1^) and β-sheet structure (1260 cm^−1^) in RSF fibers and RSF/Al_2_O_3_ NPs hybrid fibers was analyzed (Figure 3d). It was observed that RSF/Al_2_O_3_ NPs hybrid fibers with varying Al_2_O_3_ NPs content exhibited higher β-sheet structures and lower α-helix/random coil structures compared with RSF fibers, indicating that Al_2_O_3_ NPs promoted the formation of β-sheet structures in RSF/Al_2_O_3_ NPs hybrid fibers. Specifically, the content of β-sheet structure initially increased and then decreased with increasing Al_2_O_3_ NPs content, with the highest content observed in 0.8 wt% RSF/Al_2_O_3_ NPs hybrid fibers, thereby explaining the superior mechanical properties, especially breaking strength. The β-sheet is considered to be a highly ordered structure, having interactions that are arranged in an extremely organized manner to form a distribution known as the β-sheet crystal, which is widespread throughout the silk fibroin network. This unique structure effectively absorbs and cushions external stresses, thus conferring excellent mechanical properties to silk fibroin. As the content of β-sheet increases, the structure of silk fibroin fibers becomes stronger and more robust [24]. These results indicated that the integration of Al_2_O_3_ NPs accelerated the transition from the α-helix/random coil of RSF fibers to the β-sheet.

Furthermore, the crystal structure of 0.8 wt% RSF/Al_2_O_3_ NPs hybrid fibers was analyzed, as it significantly influences the mechanical properties of RSF materials [25]. WAXD revealed characteristic peaks corresponding to crystal faces (200), (210), and (002), indicating the axes a, b, and c of the crystallite, respectively (Figure 3e,f). The crystallinity of 0.8 wt% RSF/Al_2_O_3_ NPs hybrid fibers was higher than that of RSF fibers, consistent with the secondary structure results (Table 1). Moreover, the crystallite size [26] of 0.8 wt% RSF/Al_2_O_3_ NPs hybrid fibers was smaller than that of RSF fibers, suggesting that the addition of Al_2_O_3_ NPs promoted the crystallization of RSF and the formation of smaller crystallite sizes (Table 1). Nova et al. [27] considered that the ultimate strength of spider silk is controlled by the strength of β-sheet nanocrystal, and the strength of β-sheet nanocrystal is directly related to its size. The smaller the crystal inside the fiber, the better its toughness. Keten et al. [25] found that the β-crystallite size had a great influence on the mechanical properties of fibers. The larger β-crystallite in the fiber will be destroyed under lower force, while the smaller β-crystallite can provide the ability to resist deformation and fracture.

In summary, Al_2_O_3_ NPs facilitated the formation of β-sheet structures in RSF fibers, contributing to improved mechanical properties, while the addition of Al_2_O_3_ NPs enhanced the crystallinity and suppressed the crystallite size of RSF, further enhancing the mechanical strength of RSF/Al_2_O_3_ NPs hybrid fibers.

### 2.4. UV Resistance of RSF/Al_2_O_3_ NPs Hybrid Fibers

**Figure 4:**
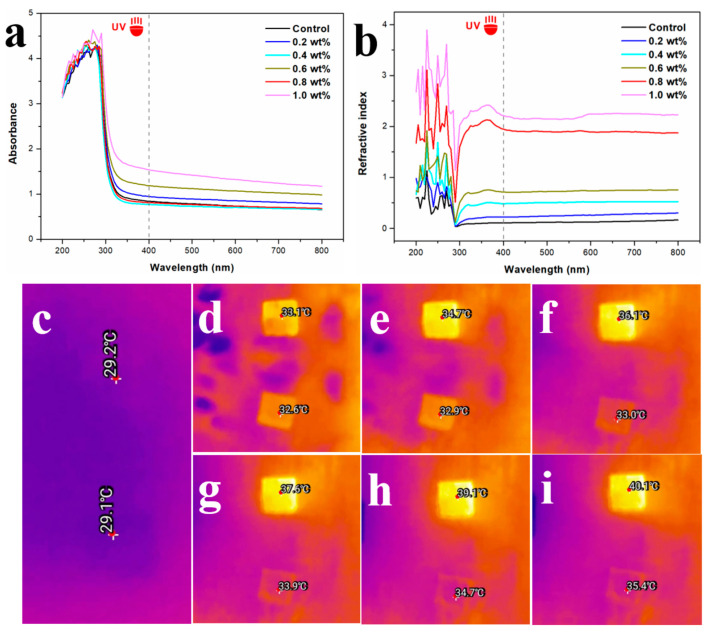
(**a**,**b**) UV absorption and refraction of RSF and RSF/Al_2_O_3_ NPs hybrid fibers with different concentrations. (**c**–**i**) Initial thermal imaging picture and thermal imaging temperature picture of 0.8 wt% RSF/Al_2_O_3_ NPs hybrid fibers in sunlight ((**d**–**i**) were real-time thermal imaging pictures when the illumination time is 1, 3, 5, 7, 10, 15 min, respectively).

The UV absorption and refractive index of 0.5 mg/mL of RSF and RSF/Al_2_O_3_ NPs solutions in the wavelength range from 200 to 800 nm are shown in Figure 4a,b. The UV absorption of RSF/Al_2_O_3_ NPs mixed solution and RSF solution is the same (Figure 4a). The UV refractive indices of the RSF/Al_2_O_3_ NPs solutions were all increased compared with the RSF solutions (Figure 4b). With the increase in Al_2_O_3_ NPs content, the UV refractive index of the RSF/Al_2_O_3_ NPs solution also increased. When the content of Al_2_O_3_ NPs was between 0.8 and 1.0 wt%, the UV refractive index of the RSF/Al_2_O_3_ NPs solution increased obviously. Combined with the mechanical properties of RSF/Al_2_O_3_ NPs hybrid fibers, 0.8 wt% Al_2_O_3_ NPs could be used as the optimal concentration of the hybrid fibers.

### 2.5. Thermal Insulation Properties of RSF/Al_2_O_3_ NPs Hybrid Fibers

Through the photothermal performance test under natural sunlight irradiation, we explored the difference in thermal insulation performance between RSF/Al_2_O_3_ NPs hybrid fibers and RSF fibers. We used an infrared imager to observe the change in fiber surface temperature for 15 min [28]. Before natural light irradiation, there was almost no difference in temperature between the blank RSF fiber and the RSF/Al_2_O_3_ NPs hybrid fiber (Figure 4c). However, after illumination, the temperature rise rate of the blank RSF fiber was faster than that of the hybrid fiber. The temperature of the blank RSF fiber increased from 29.2 °C to 36.1 °C in only 5 min and increased to 40.1 °C in 15 min (Figure 4f,i). In contrast, the temperatures of the hybrid fibers only increased to 33.0 °C and 35.4 °C, respectively. This indicates that the RSF/Al_2_O_3_ NPs hybrid fiber had better thermal insulation performance and could effectively reduce the temperature rise rate of the fiber surface. This finding provided a scientific basis for the application of the new hybrid fiber in the field of thermal insulation fabrics. 

### 2.6. Perspiration Resistance of RSF/Al_2_O_3_ NPs Hybrid Fibers

**Figure 5:**
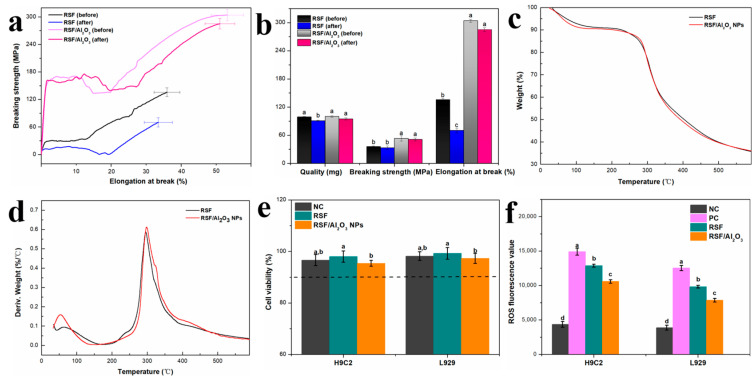
(**a**,**b**) The mechanical properties of RSF fibers and RSF/Al_2_O_3_ NPs hybrid fibers after immersion. (**c**) TG curves of RSF/Al_2_O_3_ NPs hybrid fibers and RSF fibers. (**d**) DTG curves of RSF/Al_2_O_3_ NPs hybrid fibers and RSF fibers. (**e**) Toxicity and (**f**) antioxidation of RSF/Al_2_O_3_ NPs hybrid fibers to H9C2 and L929 cells in vitro. Bars without common superscript letters differed statistically (*p* < 0.05). The experiments were repeated three times, and the results are shown as mean ± S.E.

The degradation rate and mechanical properties of RSF fibers and RSF/Al_2_O_3_ NPs hybrid fibers after wetting in alkaline simulated sweat were observed. As illustrated in Figure 5b, the degradation residual rate of RSF fiber and RSF/Al_2_O_3_ NPs hybrid fiber remained above 90%, which indicated that the damage degree of alkaline simulated sweat to RSF/Al_2_O_3_ NPs hybrid fiber was low. The low degradation rate might be due to the partial disintegration or dissolution of silk fibroin in an alkaline environment. The morphology and quality of RSF fibers and RSF/Al_2_O_3_ NPs hybrid fibers were well maintained. It could be inferred that the degree of damage to RSF/Al_2_O_3_ NPs hybrid fibers by alkaline simulated sweat was only small, indicating that RSF/Al_2_O_3_ NPs hybrid fibers had good stability and durability in alkaline environments such as sweat.

Figure 5a,b shows that the mechanical properties of RSF fibers decreased after 4 h of wetting treatment. The breaking strength and elongation at break of RSF fibers decreased by 48.26% and 6.95%, respectively, reaching 70.25 ± 4.98 MPa and 33.46 ± 3.77%, respectively. In contrast, the breaking strength and elongation at break of RSF/Al_2_O_3_ NPs hybrid fibers decreased by 6.17% and 3.97%, reaching 285.20 ± 4.33 MPa and 51.09 ± 3.23%, respectively, under the same wetting conditions. It could be seen that the mechanical properties of RSF/Al_2_O_3_ NPs hybrid fibers had very little change and higher retention. This difference might be due to the influence of the micro-alkali sweat environment. The RSF/Al_2_O_3_ NPs hybrid fibers had a higher density of crystalline regions than the RSF fibers under micro-alkali conditions, so they were not easily destroyed by the micro-alkali environment [29]. The internal crystalline region of RSF fiber contained more hydrogen bonds, which were easy to combine with alkaline substances, affecting the orderliness of the crystalline region and thus reducing the mechanical properties of RSF fiber.

For the effect of Al_2_O_3_ NPs on the thermal stability of RSF fibers, we further investigated the thermal stability of the 0.8 wt% RSF/Al_2_O_3_ NPs hybrid fibers using a thermal gravimetric analyzer (TGA). The thermogravimetric (TG) and derivative thermogravimetric (DTG) curves of the RSF fibers were obtained (Figure 5c,d), revealing similar thermal degradation processes for both fibers. As illustrated in Figure 5c,d, it demonstrated that the addition of Al_2_O_3_ NPs only had a little effect on the thermal decomposition process of RSF fibers and maintained the thermal stability of the RSF fibers. In general, RSF/Al_2_O_3_ NPs hybrid fibers showed high stability under alkaline sweat conditions, which was beneficial to the application of RSF/Al_2_O_3_ NPs hybrid fibers in the fields of heat insulation and anti-ultraviolet fabrics.

### 2.7. Cell Cytotoxicity and Antioxidant of RSF/Al_2_O_3_ NPs Hybrid Fibers

As a fiber material used to contact the skin, its biological toxicity and antioxidant properties are important indicators. In this study, we selected rat cardiomyocyte cells (H9C2) and mouse fibroblast cells (L929) as the cell models. H9C2 and L929 cells were treated with RSF and RSF/Al_2_O_3_ NPs solutions at a concentration of 0.5 mg/mL, and the cytotoxicity was evaluated by CellTiter-Blue reagent. Figure 5e shows the cytotoxicity results of RSF and RSF/Al_2_O_3_ NPs solution-treated H9C2 and L929 cells. It was observed that concentrations of 0.5 mg/mL of RSF and RSF/Al_2_O_3_ NPs solutions were non-toxic to H9C2 and L929 cells, and the cell survival rate remained above 90%. It also had a certain proliferation effect on the growth of H9C2 and L929 cells. This indicated that the RSF/Al_2_O_3_ NPs solution had good biocompatibility for H9C2 and L929 cells. Moreover, the RSF/Al_2_O_3_ NPs solution had good antioxidant properties and could effectively scavenge ROS in H9C2 and L929 cells (Figure 5f).

## 3. Experimental

### 3.1. Materials

Waste cocoons derived from *Bombyx mori* silkworm (Chinese Academy of Agricultural Sciences, Zhenjiang, China), Al_2_O_3_ nanoparticles (Jiangsu Xianfeng Nanomaterials Technology Co., Ltd., Nanjing, China), absolute ethanol (C_2_H_5_OH) (Sinopharm Group Chemical Reagent Co., Ltd., Beijing, China), sodium carbonate (Na_2_CO_3_) (Sinopharm Group Chemical Reagent Co., Ltd., Beijing, China), Formic acid (FA) (Shanghai Aladdin Co., Ltd., Shanghai, China), and anhydrous calcium chloride (CaCl_2_) (Sinopharm Group Chemical Reagent Co., Ltd., Beijing, China).

### 3.2. Preparation of RSF Solution

Add an appropriate amount of silk to a 0.05% Na_2_CO_3_ aqueous solution. Boil for 30 min at a bath ratio of 1:20. Rinse 3 times with ultrapure water at 60 °C to remove impurities and residual ions. Repeat this step 3 times. Finally, the degummed silk was dried to constant weight at 45 °C. The regenerated RSF solution was prepared by dissolving the degummed silk in 5% CaCl_2_-FA solution. Different masses of Al_2_O_3_ NPs were added to the RSF solution, and the mass fraction of the degummed silk was 0.2%, 0.4%, 0.6%, 0.8%, and 1%. The reaction was stirred at room temperature for 4 h, and the spinning solution was obtained after stirring evenly.

### 3.3. Preparation of RSF/Al_2_O_3_ NPs Hybrid Fiber

Using a self-made wet spinning device, we inject the spinning solution into the coagulation tank through a medical syringe. The solidification groove was 1 m, and the solidification liquid was 75% ethanol. The spinning solution enters the coagulation solution at a speed of 2 mL/h through an injection pump with a spinning needle diameter of 0.21 mm. The spinning solution rapidly condenses into uniform filamentous fibers in the coagulation solution. The as-spun fibers were collected on the rotating shaft after stretching treatment. We placed the collected RSF fibers in 75% ethanol solution for 2 h to remove the residual solvent and finalize the design. Finally, the RSF fiber was taken out and naturally air-dried for 24 h.

### 3.4. Characterization of RSF/Al_2_O_3_ NPs Hybrid Fiber

The morphologies of RSF/Al_2_O_3_ NPs hybrid fibers were observed using field emission scanning electron microscopy (SEM) (JSM-IT500HR, Tokyo, Japan) at 20 kV after coating with gold using a sputter coater (SBC-12, Beijing, China). Fourier transform infrared spectroscopy (FTIR): The structural changes of the fibers were analyzed by fourier transform infrared spectrometer (Nicolet IS 10, ATR, Waltham, MA, USA) of Thermo Scientific (Waltham, MA, USA), with a spectral range of 400–4000 cm^−1^ and a spectral resolution of 0.4 cm^−1^. Wide-angle X-ray diffraction (WAXD) spectrum test: The fiber’s structure was analyzed using WAXD (Rigaku TTR-III, Tokyo, Japan) with a copper target (CuKα, λ = 0.1542) at 40 KV. Spectra were fitted using Peakfit software, and parameters such as crystallinity and crystallite size of the fibers were calculated via semi-quantitative analysis. Thermal deterioration was assessed using a thermal gravimetric analyzer (TGA) (Q5000, TA Instruments, New Castle, DE, USA), with heating performed at a rate of 10 °C/min from ambient to 600 °C in N_2_. Both DTG and TG curves were recorded. The results were plotted using Origin. 

### 3.5. Mechanical Property Measurement

Before performing a mechanical property analysis, we need to exclude samples that have broken or slipped out of the clamp before the test, as these samples cannot be further evaluated. To test the mechanical properties of silk fibroin fibers reinforced by Al_2_O_3_ NPs with different mass concentrations, we used a single-fiber mechanical tensile tester (Instron 3343, Norwood, MA, USA). The temperature of the test environment was controlled at 24 ± 0.5 °C, and the relative humidity was 42 ± 5%. In the tensile test, we used a tensile rate of 2 mm/min and a gauge length of 6 mm. In order to improve the reliability of the test results, we selected 15 fibers for each group of samples for tensile testing and took the average as the final result.

### 3.6. Determination of Sweat Resistance and Cytotoxicity

According to the requirements of GB/T3922-2013 standard [30], we tested the sweat resistance of regenerated silk fiber. The sweat used in the test was configured by the following methods: First, 0.5 g C_6_H_9_N_3_O_2_·HCl·H_2_O, 5 g NaCl, and 5 g Na_2_HPO_4_·12H_2_O were taken and added to 1000 mL of deionized water. Then dilute sodium hydroxide was added to adjust the pH of sweat to 8.0 to simulate alkaline sweat. In the test, three groups of 50 mg RSF fibers and RSF/Al_2_O_3_ NPs hybrid fibers were taken and placed in alkaline sweat at a bath ratio of 1:50. Next, we cultured at 37 °C for 4 h. After the culture, we washed the silk fibroin fiber with deionized water and dried it in an oven at 40 °C. Finally, we measured the quality and mechanical properties of RSF fibers [31].

Rat cardiomyocyte cells (H9C2) and mouse fibroblast cells (L929) were seeded in a 96-well cell culture plate and cultured for 24 h. The RSF/Al_2_O_3_ NPs (1000 μg/mL) solutions were diluted to 1/10 of original concentration with DMEM. Each diluted solution was added to 96-well plate (90 μL/well) for certain amount of time (2D culture model for 24 h, 3D culture model for 72 h). Finally, 10 μL of CellTiter-Blue reagent was added to each well for 3 h incubation. The plates were then read to collect the fluorescence absorbance of 560 nm by a SynergyTM H1 microplate reader (Bio Tek Inc., Winooski, VT, USA). The relative survival rate of cells (%) = (OD_samples_/OD_control_) × 100. At the same time, the growth of cells was observed by cell imaging system. The ROS clearance rate of the sample in the cell was detected by Beyotime ROS detection kit. The specific experimental steps were in accordance with the instructions of the kit, and the aseptic operation was ensured.

### 3.7. Determination of Anti-Ultraviolet and Thermal Insulation Properties

The UV-2600 (Shimadzu, Kyoto, Japan) ultraviolet spectrophotometer was used to measure the UV absorbance and reflectivity of different concentrations of regenerated silk fibroin composite solutions. The calcium chloride formate solution was used as the baseline for measurement, and the UV absorbance and reflectivity of the regenerated silk fibroin solution dissolved in calcium chloride formate were used as the blank control [32].

The fabric sample and the blank sample were placed under the same natural sunlight conditions to ensure that both could be fully irradiated by light. The infrared temperature camera (Flir, Boston, MA, USA) is placed at a certain height. Ensure that the temperature of the sample can be accurately measured. The initial temperatures of the fabric sample and the paper sample were photographed and recorded. The temperature of the fabric sample and the blank was recorded at a fixed time. Continuously observe and record the temperature until it reaches a certain time point or the temperature rise tends to be stable, and compare the temperature change curve of the fabric sample and the blank, especially the difference in the heating rate [33].

## 4. Conclusions

In this study, RSF/Al_2_O_3_ NPs hybrid fibers with high strength, heat insulation, and UV resistance were prepared by wet spinning. The addition of Al_2_O_3_ NPs could enhance the mechanical properties of RSF/Al_2_O_3_ NPs hybrid fibers. The mechanical properties of 0.8 wt% RSF/Al_2_O_3_ NPs hybrid fibers were the best. The appropriate amount of Al_2_O_3_ NPs could increase the β-sheet content of RSF/Al_2_O_3_ NPs hybrid fibers. Moreover, RSF/Al_2_O_3_ NPs hybrid fibers and fabrics had excellent heat insulation and UV resistance. Thus, the data from this study on Al_2_O_3_ NPs in enhanced RSF are expected to be helpful in the future for the design and production of more innovative functional fibers. Overall, the present research provides a method for manufacturing fibers with ultraviolet resistance and thermal insulation capabilities that can be used in multifunctional fibers.

## Figures and Tables

**Table 1:** The crystallinity and crystallite size of 0.8 wt% RSF/Al_2_O_3_ NPs hybrid fibers.

Samples	Crystallite Size (nm)				
	a	b	c	V/nm^3^	Crystallinity
RSF fibers	1.82	2.37	2.09	9.02	40.61%
0.8 wt% RSF/Al_2_O_3_ NPs hybrid fibers	1.83	2.42	1.71	7.57	41.85%

## Data Availability

The data presented in this study are available on request from the corresponding author.

## References

[1] Zhang J.R., Sun J., Li B., Yang C.J., Shen J.L., Wang N., Gu R., Wang D.G., Chen D., Hu H.G. (2020). Robust biological fibers based on widely available proteins: Facile fabrication and suturing application. Small.

[2] Guo J.J., Jia L.L., Fometu S.S., Ma Q., Wang J.J., Li H., Jiang L., Wu G.H. (2022). Fabrication of High Toughness Silk Fibroin/Tungsten Disulfide Nanoparticles Hybrid Fiber and Self-Heating Textile by Wet Spinning. J. Renew. Mater..

[3] Guo J.J., Yang B., Ma Q., Fomentu S.S., Wu G.H. (2021). Photothermal Regenerated Fibers with Enhanced Toughness: Silk Fibroin/MoS_2_ Nanoparticles. Polymers.

[4] Wang Z.Q., Yang H.W., Li Y., Zheng X.H. (2020). Robust silk fibroin/graphene oxide aerogel fiber for radiative heating textiles. ACS Appl. Mater. Interfaces.

[5] Cai H.H., Gao L., Chen L.Z., Chen X., Liu Z.L., Li Z., Dai F.Y. (2022). An effective, low-cost and eco-friendly method for preparing UV resistant silk fabric. J. Nat. Fibers.

[6] Wang S.D., Wang K., Ma Q., Qu C.X. (2020). Fabrication of the multifunctional durable silk fabric with synthesized graphene oxide nanosheets. Mater. Today Commun..

[7] Abo El-Ola S.M., Kotb R.M., Shaker R.N. (2021). Photocatalytic finishing of silk and viscose fabrics. J. Text. Ins..

[8] Gao L.Z., Bao Y., Cai H.H., Zhang A.P., Ma Y., Tong X.L., Li Z., Dai F.Y. (2020). Multifunctional silk fabric via surface modification of nano-SiO_2_. Text. Res. J..

[9] Subburaayasaran A.S., Sampath Kumar K.S., Kumar D.V., Ramachandran T., Prakash C., Vijayakumar H.L. (2022). Comparative Studies on Thermal Comfort Properties of Eri Silk, Wool/Eri Silk, Cotton, and Micro-denier Acrylic Double-layered Knitted Fabrics. J. Nat. Fibers.

[10] Zhou B., Wang H.L., Zhou H.T., Wang K., Wang S.D. (2020). Natural flat cocoon materials constructed by eri silkworm with high strength and excellent anti-ultraviolet performance. J. Eng. Fibers Fabr..

[11] Gao Y., Jiang M.W., Bao L.Z., Yin Z.C., Zhang J.C., Pan G.Z., Zheng S.A., Zheng Y.F., Wu C.J., Li M. (2022). Preparation and characterization of silk fibroin/silica thermal insulation coatings on catheters. Surf. Innov..

[12] Nayar P., Waghmare S., Singh P., Najar M., Puttewar S., Agnihotri A. (2020). Comparative study of phase transformation of Al_2_O_3_ nanoparticles prepared by chemical precipitation and sol-gel auto combustion methods. Mater. Today Proc..

[13] Deng Y., Li J.H., Deng Y.X., Nian H.E., Jiang H. (2018). Supercooling suppression and thermal conductivity enhancement of Na_2_HPO_4_·12H_2_O/expanded vermiculite form-stable composite phase change materials with alumina for heat storage. ACS Sustain. Chem. Eng..

[14] Gong S.Q., Cheng X.M., Li Y.Y., Shi D.W., Wang X.L., Zhong H. (2019). Enhancement of ceramic foam modified hierarchical Al_2_O_3_@expanded graphite on thermal properties of 1-octadecanol phase change materials. J. Energy Storage.

[15] Xu H., Zhang C.M., Cai J.X., Wang J., Liu K.Q., Cheng X.W. (2022). Synthesis and characterization of activated alumina with high thermal stability by a low-heat solid-phase precursor method. Microporous Mesoporous Mater..

[16] Ghanizadeh S., Bao X.J., Vaidhyanathan B., Binner J. (2014). Synthesis of nano α-alumina powders using hydrothermal and precipitation routes: A comparative study. Ceram. Int..

[17] Fu L.P., Huang A., Gu H.Z., Ni H.W. (2018). Properties and microstructures of lightweight alumina containing different types of nano-alumina. Ceram. Int..

[18] Ji Q.Y., Zhang L., Jiao X.L., Chen D.R. (2023). Alpha Al_2_O_3_ Nanosheet-Based Biphasic Aerogels with High-Temperature Resistance up to 1600 °C. ACS Appl. Mater. Interfaces.

[19] Korkmaz N., Aksoy S.A. (2016). Enhancing the performance properties of ester-cross-linked cotton fabrics using Al_2_O_3_-NPs. Text. Res. J..

[20] Zheng K.S., Zhang Z.P., Wang X.M., Zhan R.Z., Chen H.J., Deng S.Z., Xu N.S., Chen J. (2019). Mechanism of photoluminescence quenching in visible and ultraviolet emissions of ZnO nanowires decorated with gold nanoparticles. Jpn. J. Appl. Phys..

[21] Guo J.J., Xu C., Yang B., Li H., Wu G.H. (2023). The size effect of silver nanoparticles on reinforcing the mechanical properties of regenerated fibers. Molecules.

[22] Ling S.J., Qi Z.M., Knight D.P., Shao Z.Z., Chen X. (2011). Synchrotron FTIR microspectroscopy of single natural silk fibers. Biomacromolecules.

[23] Yazawa K., Hidaka K. (2020). Pressure-and humidity-induced structural transition of silk fibroin. Polymer.

[24] Lin N., Cao L., Huang Q., Wang C., Wang Y., Zhou J., Liu X.Y. (2016). Functionalization of silk fibroin materials at mesoscale. Adv. Funct. Mater..

[25] Keten S., Xu Z., Ihle B., Buehler M.J. (2010). Nanoconfinement controls stiffness, strength and mechanical toughness of β-sheet crystals in silk. Nat. Mater..

[26] Fang G., Zheng Z., Yao J., Chen M., Tang Y., Zhong J., Qi Z., Li Z., Shao Z., Chen X. (2015). Tough protein-carbon nanotube hybrid fibers comparable to natural spider silks. J. Mater. Chem. B.

[27] Nova A., Keten S., Pugno N.M., Redaelli A., Buehler M.J. (2010). Molecular and nanostructural mechanisms of deformation strength and toughness of spider silk fibril. Nano Lett..

[28] Ma H., Zhao H., Li X., Hou K., Wang J., Cai Z. (2023). Double-sided functional infrared camouflage flexible composite fabric for thermal management. Ceram. Int..

[29] Hao X.Y., Wang X., Yang W.M., Ran J.B., Ni F.F., Tong T., Dai W., Zhang L.Y., Shen X.Y., Tong H. (2019). Comparisons of the restoring and reinforcement effects of carboxymethyl chitosan-silk fibroin (Bombyx Mori/Antheraea Yamamai/Tussah) on aged historic silk. Int. J. Biol. Macromol..

[30] (2013). Textiles—Tests for Colour Fastness—Colour Fastness to Perspiration.

[31] Holland C., Hawkins N., Frydrych M., Laity P., Porter D., Vollrath F. (2019). Differential scanning calorimetry of native silk feedstock. Macromol. Biosci..

[32] Wang X., Sun X.T., Guan X.Y., Wang Y.Q., Chen X.G., Liu X.Q. (2021). Tannic interfacial linkage within ZnO-loaded fabrics for durable UV-blocking applications. Appl. Surf. Sci..

[33] Cui Y., Gong H.X., Wang Y.J., Li D.W., Bai H. (2018). A thermally insulating textile inspired by polar bear hair. Adv. Mater..

